# X-Ray CT Phenotyping Reveals Bi-Phasic Growth Phases of Potato Tubers Exposed to Combined Abiotic Stress

**DOI:** 10.3389/fpls.2021.613108

**Published:** 2021-03-30

**Authors:** Jessica K. Van Harsselaar, Joelle Claußen, Jens Lübeck, Norbert Wörlein, Norman Uhlmann, Uwe Sonnewald, Stefan Gerth

**Affiliations:** ^1^Department of Biology, Friedrich-Alexander-University Erlangen-Nuremberg, Erlangen, Germany; ^2^Fraunhofer Institute for Integrated Circuits IIS, Development Centre X-Ray Technology, Fürth, Germany; ^3^School of Agriculture, Food and Wine, The University of Adelaide, Adelaide, SA, Australia; ^4^Solana Research GmbH, Windeby, Germany

**Keywords:** X-ray, belowground, phenotyping, tuber (potato), genetic diversity, biomass, non-invasive (non-contact) measurements, abiotic stress

## Abstract

As a consequence of climate change, heat waves in combination with extended drought periods will be an increasing threat to crop yield. Therefore, breeding stress tolerant crop plants is an urgent need. Breeding for stress tolerance has benefited from large scale phenotyping, enabling non-invasive, continuous monitoring of plant growth. In case of potato, this is compromised by the fact that tubers grow belowground, making phenotyping of tuber development a challenging task. To determine the growth dynamics of tubers before, during and after stress treatment is nearly impossible with traditional destructive harvesting approaches. In contrast, X-ray Computed Tomography (CT) offers the opportunity to access belowground growth processes. In this study, potato tuber development from initiation until harvest was monitored by CT analysis for five different genotypes under stress conditions. Tuber growth was monitored three times per week *via* CT analysis. Stress treatment was started when all plants exhibited detectable tubers. Combined heat and drought stress was applied by increasing growth temperature for 2 weeks and simultaneously decreasing daily water supply. CT analysis revealed that tuber growth is inhibited under stress within a week and can resume after the stress has been terminated. After cessation of stress, tubers started growing again and were only slightly and insignificantly smaller than control tubers at the end of the experimental period. These growth characteristics were accompanied by corresponding changes in gene expression and activity of enzymes relevant for starch metabolism which is the driving force for tuber growth. Gene expression and activity of Sucrose Synthase (SuSy) reaffirmed the detrimental impact of the stress on starch biosynthesis. Perception of the stress treatment by the tubers was confirmed by gene expression analysis of potential stress marker genes whose applicability for potato tubers is further discussed. We established a semi-automatic imaging pipeline to analyze potato tuber delevopment in a medium thoughput (5 min per pot). The imaging pipeline presented here can be scaled up to be used in high-throughput phenotyping systems. However, the combination with automated data processing is the key to generate objective data accelerating breeding efforts to improve abiotic stress tolerance of potato genotypes.

## Introduction

Independent climate change models predict that global temperatures will increase, and patterns of rainfall will change entailing periods of drought on the one hand and floods on the other hand (Cook et al., 2007). As a result, plants will be—and are already—exposed to changing environmental conditions which cause substantial yield losses (Hijmans, 2003; Ciais et al., 2005). The prevention of such losses is of particular importance regarding the rapidly growing world population and the increasing need for food and feed (Alexandratos and Bruinsma, 2012). Potato (*Solanum tuberosum* L.) is the fourth most important crop plant worldwide and as such of great significance with respect to food security (Thiele et al., 2010). Potato productivity is limited by abiotic stresses like drought and heat (Aksoy et al., 2015). Moreover, potato plants are cultivated worldwide including geographic areas that are prone to heat waves and drought periods. Therefore, potato yield is likely to be negatively affected by these stress factors alone or in combination.

Originating from the temperate zones of the Andes, potato plants prefer cooler temperatures below 20°C and a short photoperiod (12 h) (Van Dam et al., 1996). Potato tubers are formed by longitudinal cell division in pith and cortex from underground stem-derived shoots called stolons. Tuber formation is associated with increased cell expansion and division and an enhanced starch and storage protein biosynthesis and deposition. Sucrose unloading changes from apoplasmic to symplasmic (Viola et al., 2001) rendering Sucrose Synthase (SuSy) as the main enzyme hydrolyzing sucrose and thus providing building blocks for other metabolic processes like starch biosynthesis (Appeldoorn et al., 1997). As a consequence, SuSy has been identified as a determinant of sink strength (Zrenner et al., 1995). Both, heat and drought, have been shown to inhibit tuberization causing decreased tuber number, size and quality (Levy, 1985; Deblonde and Ledent, 2001). These adverse effects are caused by an interference of heat and drought with the formation of the tuberization signal SP6A (Navarro et al., 2011; Hastilestari et al., 2018), carbon allocation to developing tubers (Wolf et al., 1990; Gawronska et al., 1992), and tuber filling (Krauss and Marschner, 1984). In addition, starch mobilization has been described during both, heat and drought stress, leading to increased reducing sugar content of the tubers (Dahal et al., 2019).

Conventional methods to follow tuber growth over time involved the removal of tubers and/or alterations in the substrate leading to potential disturbances of source-sink relationships (Pérez-Torres et al., 2015). X-ray computed tomography (CT) is used to determine non-destructively and non-invasively above ground plant material for example of wheat ears (Hughes et al., 2017; Schmidt et al., 2020), rice tillers (Yang et al., 2011), or seeds (Duong Quoc Le et al., 2019). For belowground it is used to observe the soil root interactions (Rogers et al., 2016), the root systems (Tracy et al., 2010; Mooney et al., 2012; Zappala et al., 2013; Metzner et al., 2015; Pfeifer et al., 2015; Gao et al., 2019; Teramoto et al., 2020) or other plant organs like cassava or potato tubers (Pérez-Torres et al., 2015). Other methods to observe tuber growth dynamics non-destructively and non-invasively are magnetic resonance imaging (MRI) (Metzner et al., 2015; van Dusschoten et al., 2016) and ground penetrating radar (GPR) (Delgado et al., 2017). CT has previously been shown to allow non-invasive tracking of tuber growth and reproducible determination of tuber volume (Ferreira et al., 2010). A hindrance of this method was the high demand for manual corrections of the segmentation of the images which led to low sample throughput capacities, time-consuming data analysis and high costs.

Here we describe the implementation of a medium-throughput imaging pipeline enabling us to continuously monitor growth of potato tubers by means of CT analysis. We observed the implications of combined heat and drought stress on tuber growth velocity *in vivo* and analyzed gene expression of potato tubers with destructive sampling.

## Materials and Methods

### Plant Material and Growth Conditions

*Solanum tuberosum* plantlets of the cultivars Agria, Saturna, Tomensa, and Ramses were obtained from Solana Research GmbH (Windeby, Germany). The cultivar Diamant was obtained from KWS Potato BV (Nagele, Netherlands). All plantlets were propagated in tissue culture on MS-Medium (Murashige and Skoog, 1962) containing 2% (w/v) sucrose under conditions of 16 h light (150 μmol m^–2^ s^–1^) and 8 h dark at 21°C. Plants were transferred to individual pots with 15 cm diameter and a volume of 1.5 L containing sieved soil (Einheitserde Classic ED73; sieve grid 0.5 cm). The pots were placed in plant growth chambers (Conviron, Winnipeg, Canada) under conditions of 16 h light at 21°C and 8 h dark at 18°C and 50% humidity during the day and 35% humidity at night. Plants were watered daily with 50 ml per day/plant and tuber growth was monitored by CT three times per week. In the first experiment, four plants each of the cultivars Agria, Saturna, Tomensa, Ramses and Diamant were monitored at the same time. When tubers were detectable *via* CT, combined heat and drought stress was applied by increasing the temperature to 29°C during the light period and 21°C during the dark period and reducing the amount of water given to each plant from 50 to 30 ml/day. In the second experiment with the cultivar Diamant, 30 plants were used of which 16 were monitored by CT analysis. After tuber initiation, as determined by CT monitoring, drought and mild heat stresses were applied to half of the plants for 2 weeks while the other half of the plants served as control group. The control group was watered with 50 ml per day/plant for the whole time while the stress group was subjected to the conditions described above.

### X-Ray CT Imaging

**FIGURE 1:**
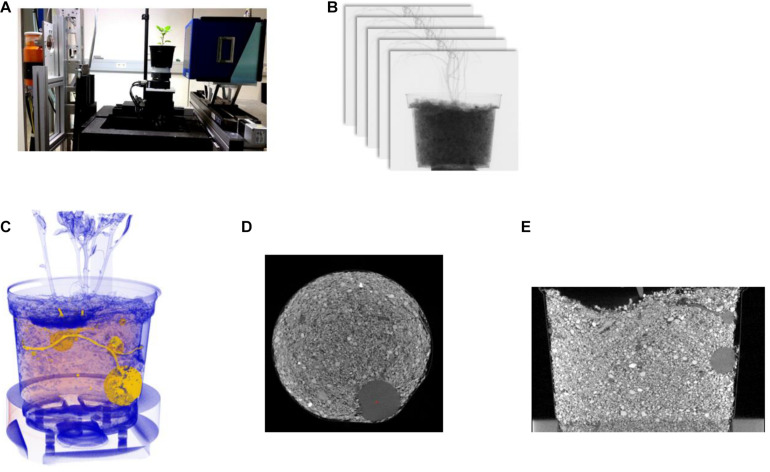
Panel **(A)** shows a potato plant in the CT-System. On the left-hand side is the X-ray tube and on the right-hand side the detector, which collects the projections; panel **(B)** represents the stack of 2D projections; panel **(C)** shows a 3D visualization of the reconstructed and segmented data; **(D)** horizontal and **(E)** the vertical cross-section.

All plants were measured at an individually designed CT system at the Fraunhofer EZRT in Fuerth, Germany using a GE 225 MM2/HP source, Aerotech axis systems and the Meomed XEye 2020 Detector operating with a binned rectangular pixel size of 100 μm (see Figure 1A). The source was operated at 175 kV acceleration voltage with a current of 4.7 mA, recording 800 individual projections with an integration time of 380 ms per projection. The stack of projections was made with continuous sample rotation (fly-by) over 360°, resulting in a measurement time of 5 min per pot. To harden the x-ray spectra, a 1 mm thick copper pre-filtering was applied with the filter mounted directly in front of the source. For the reconstruction of the recorded projection stack (see Figures 1B,C), we used a filtered back projection reconstruction library from Fraunhofer EZRT. The algorithms are implemented following the description of Buzug (2008). For a stable analysis result, we normalized all reconstructed volumes regarding the unattenuated intensity of areas without any transmitted volume. Thus, the resulting gray-scale range is comparable throughout the whole series of measurements. In the CT system we used a focus object distance of 725 mm and a focus detector distance of 827 mm. This resulted in a reconstructed voxel size of 88.9 μm. With this small distance between the pot and the detector, the resolution is mainly determined from the rectangular pixel size of the detector and not influenced from the focal spot size of the x-ray source. Thus, a scanning time of 5 min per pot is feasible. The loading of the potato pots was done manually (see Figure 1A). Figures 1D,E shows a horizontal and a vertical cross-section of the reconstructed volume, respectively.

### Implementation of an Automated Data Analysis Platform for CT Imaging

**FIGURE 2:**
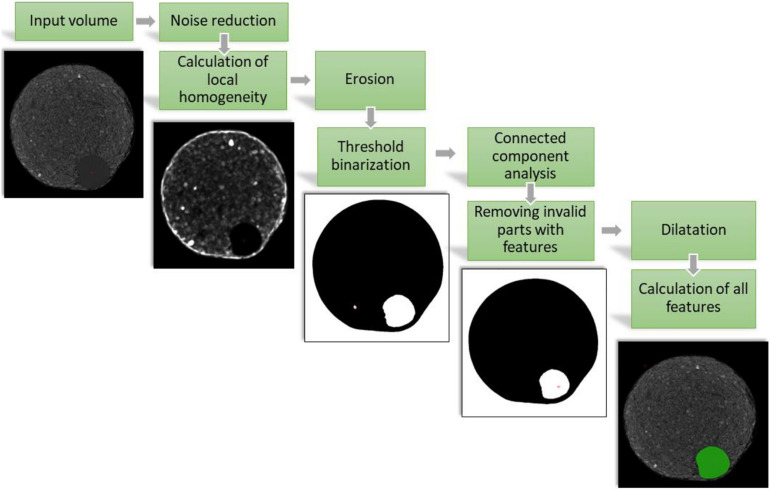
Single steps of the image processing for segmenting potato tubers and the final segmentation as green overlay over the original volume. For representation virtual 2D cross sections at the same depth of the reconstructed 3D volume of the pot are shown.

We generated an automated data analysis platform for the high-throughput CT analysis. For better performance the image processing was done in a special C++ application which can be used in the VolumePlayerPlus visualization and segmentation software. To analyze all plants in a comparable manner, special segmentation algorithms were used. Only using the reconstructed linear absorption coefficient, results in many false positives and it would be very hard to distinguish tubers from different plant material inside the soil (like turf, stolon and roots). Thus, we used the hypothesis that the soil is inhomogeneous compared to tubers for our image segmentation pipeline which contains eight steps (see Figure 2).

As first step after the reconstruction we applied a size adaptable median filter in 3D to reduce the noise. On this filtered volume we calculated pixel wise the local variance:

(1) Vl⁢o⁢c⁢a⁢l⁢(x)=12⁢π⁢λ2⁢exp⁢(-(x2-x02)22⁢λ2)-(12πλ2⁢exp⁡(-(x-x0)22λ2))2.

Now, compared to the soil, tubers are more homogeneous and a single threshold binarization separates most of the surrounding soil from the tubers. However, in this state potato tubers and stolons are still connected. Therefore, we applied a morphological operator called Erosion (Soille, 1998). As next step we calculated the connected components with a chessboard metric to have each object in the volume individually labeled and applied a morphological operator called Dilatation. This was necessary because the local variance calculation and the erosion reduced the size of the labeled segments. With the Dilatation we reversed this reduction and received the original size of the tubers.

At this stage only homogenously labeled objects were left over in the reconstructed dataset. For all of these objects, parameters like the volume, the mean absorption coefficient, the position and the aspect ratio were calculated. Scanning the same pot several times in a row, allowed to further differentiate between tubers and other false positive segmented homogenous objects like stones and parts of unconnected stolons. A tuber has to grow over time and the mean reconstructed absorption coefficient is depending on the material and density (Buzug, 2008), thus, it is possible to distinguish homogenous clay parts from the tubers. In case the aspect ratio is very different from a sphere all the disconnected homogenous stolons can be filtered as well. To track the potato tubers over time we calculated the center of mass of the individual potato tuber and compared it with the position of the timepoint before. The maximal distance was determined by the diameter of the sphere equivalent volume of the potato tuber at this timepoint. This resulted in a time dependent threshold of maximal difference between the center of mass of two timepoints.

Using these algorithms, it is possible to reconstruct, segment and analyze all plants at each measured time point with only one set of parameters. Both experiments combined 592 measurements in a total of 36 plants. An evaluation version of the Tuber segmentation package as well as some reference volumes can be obtained by the senior author (SG).

### Sampling of Tuber Material

Tuber samples from the experiment with the cultivar Diamant were taken at three time points; (1) 8 days after initialization of the stress period, when the tubers had stopped growing (TP1), (2) 3 days after the stress period, when the tubers had started growing again (TP2), and (3) at the end of the experimental period, 2 weeks after the end of the stress phase (TP3). At each time point five plants per treatment were harvested and the leaf and tuber biomass were measured with a laboratory balance. At the first time-point, 10 plants (five per condition) were harvested which had not been monitored by CT analysis. At the second time-point, two plants per condition had not been subjected to CT analysis, while the other three sampled plants had been monitored. At the end of the experimental period the remaining 10 plants which had been monitored *via* CT imaging were harvested and biomass was determined. At each time point and condition, 10 tubers were selected for sampling. Samples were immediately frozen in liquid nitrogen and stored at −80°C until further use.

### Determination of Starch Content

Starch was quantified in tuber samples extracted with 80% ethanol incubated for 1 h at 80°C. After centrifugation, removal of the supernatant, and washing with ethanol and water, 0.2 M Hepes-KOH, pH 7.5 was added and samples kept at 4°C overnight. After heating the samples to 95°C for 1.5 h, the pH was neutralized with 1 N acetic acid. Next, starch extracts were incubated with Amyloglucosidase overnight at 55°C and pH 5.5. The spectrophotometric determination of released glucose was conducted as described previously (ap Rees et al., 1977).

### Measurement of Sucrose Synthase Activity

Sucrose Synthase activity was measured according to Zrenner et al. (1995). Protein content was determined according to Bradford (1976).

### RNA Isolation

RNA was isolated as described previously (Logemann et al., 1987). Total RNA was quantified, and quality controlled using the ND-1000 Spectrophotometer (NanoDrop Technologies).

### CopyDNA Synthesis and Quantitative Reverse Transcriptase Polymerase Chain Reaction Analysis

Two μg of total RNA were treated with DNase I (Thermo Scientific) prior to reverse transcription using oligo d(T) primers and RevertAid^TM^ H minus first strand copyDNA (cDNA) synthesis kit (Thermo Scientific) according to the manufacturer’s instructions. For relative quantification of starch gene derived transcripts, quantitative reverse transcriptase polymerase chain reaction (qRT-PCR) analyses were performed using the AriaMX qPCR system (Agilent Technologies) in combination with the Brilliant II SYBR^®^ Green QPCR Master Mix (Agilent Technologies) with four biological replicates for each condition and two technical replicates. Ubi3 [L22576, (Kloosterman et al., 2005)] expression was used for normalization of target gene expression. The thermal profile was as follows: 1 cycle 10 min at 95°C for DNA polymerase activation followed by 40 cycles of 30 s at 95°C, 30 s 60°C and 30 s 72°C and subsequently a melting curve. Primers were designed using the Primer-designing tool on the NCBI website (Ye et al., 2012) to have a product length ranging from 70–150 bp and a melting temperature from 59–61°C. Target genes and sequences are listed in Table 1.

**TABLE 1:** Target genes and primer sequences for qRT-PCR analyses.

Target gene	Forward primer sequence (5′–3′)	Reverse primer sequence (5′–3′)
Ubiquitin (ubi3, L22576)	TTCCGACACCATCGACAATGT	CGACCATCCTCAAGCTGCTT
Drought-induced 19 (DI19, PGSC0003DMT400011781)	CCAGTGCAGATCCTGATCCC	GCGCTTTCTTGTGTTGAGCA
Lipoxygenase 1 (LOX1, Sotub01g036960.1.1)	CAGAGCCAGGAAGTGCAGAG	TGAATCATTCTGCCCCAGGTAA
Abscisic acid and environmental stress-inducible protein (TAS14, PGSC0003DMT400009069)	TAACACCTGTTGTGCCTCCA	CTTGGTTGCCGTATTGTGCC
Heat-shock protein (HSP, PGSC0003DMT400032851)	GAAACACCTCAAGCTCATTGC	TCTTCTGCTTTCCACTTTCCA
Sucrose Synthase 4 (SUSY4, PGSC0003DMT400007506)	ATGAACCGAGTGAGGAATGG	GCTGGACCACCGTGATTAGT
Granule-bound starch synthase (GBSS, PGSC0003DMT400031568)	CTCACACAGCTCAACAAGTGC	GTGAAGCTGTGATGCTTGCC
Glucose-phosphate Translocator 2.1 (GPT2.1, PGSC0003DMT400013500)	TGGCTGCTGGCTCTCTTATG	TGAGCCACAGCAACAGGAAA

## Results

### Calibration of Tuber Fresh Weight From CT Analysis

**FIGURE 3:**
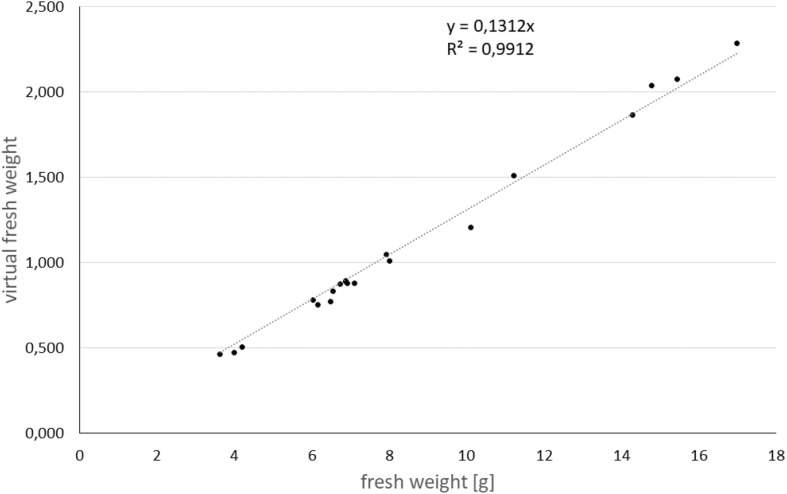
Calibration curve for virtual tuber fresh weight to real tuber fresh weight.

Analyzing the volume and the mean linear absorption coefficient for each tuber allows to calculate a virtual fresh weight. For this assumption we used the high correlation between the linear absorption coefficients of the reconstruction of the 3D volume with the physical density of the object. However, this only holds, as long as the elemental composition of the object of interest is more or less constant. In case of potato tubers, the elemental composition is mainly determined by hydrocarbon chains and water. In Figure 3, the correlation of the fresh weight with the non-destructively determined virtual fresh weight for individual potato tubers was calculated with an R^2^ of 0.99.

Applying this as calibration, it was possible to directly calculate the corresponding fresh weight during the growth process for each measured tuber. With the results of the depicted image processing pipeline and the corresponding calculated features, a python script collected each single result and calculated the growth statistics over time for each potato tuber and variety.

### Growth Curves of Different Potato Varieties as Result of the Imaging Pipeline

**FIGURE 4:**
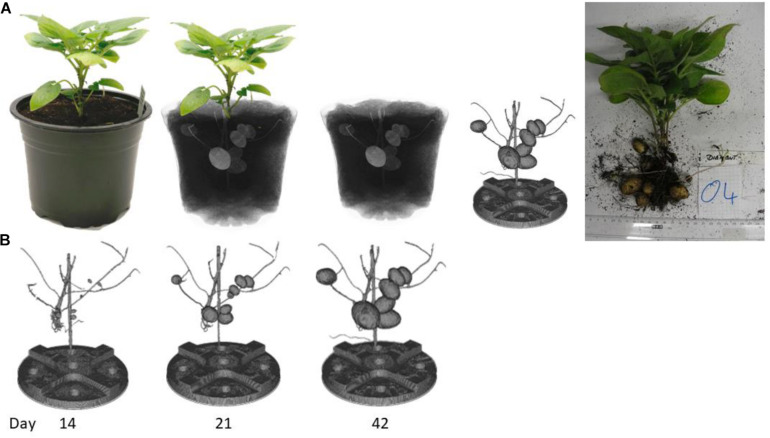
Schematic representation of the virtual excavation of potato tubers using the automated segmentation process. The scanned plant is reconstructed after the CT measurement and virtually excavated. **(A)** On the right-hand side, the excavated tubers are shown after the last measurement time point. **(B)** three timepoints as example of the growth of the potato tubers.

Potato plants were monitored *via* CT imaging every second day. After the measurement the potato tubers were virtually excavated out of the soil for each time point. This segmentation process was automated and ran with the same parameter for all plants even if there were differences in potato tuber sizes or in soil moisture due to stress conditions. Figure 4A demonstrates the segmentation process from plant to segmented tubers. Figure 4B depicts some timepoints of the growth of the potato tubers. The image from day 14 shows that the potato tubers can be segmented from the beginning where the potato tubers were only 3.6 mm in diameter. The image of day 42 demonstrates that even if there were several bigger potato tubers close together, the segmentation is possible. On the segmented tubers the volume and fresh weight of each tuber for each time point was calculated to determine the growth curve over time. Thus, each potato tuber could be tracked over the different measurements and even with slight variation in moisture content of the soil robust tuber tracking and segmentation was possible.

**FIGURE 5:**
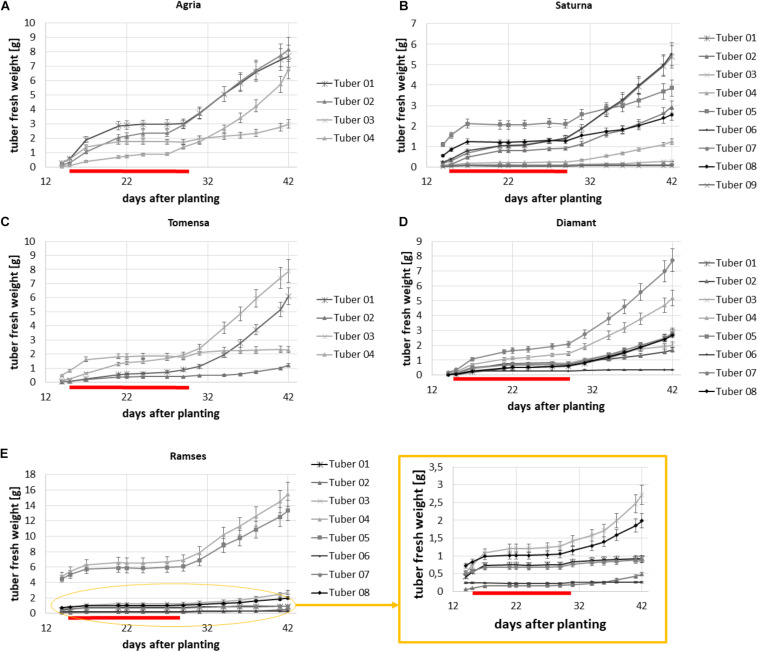
Growth over time for each tuber of one exemplary plant for five different genotypes. **(A)** Agria, **(B)** Saturna, **(C)** Tomensa, **(D)** Diamant, and **(E)** Ramses where growth curves of tubers in the yellow circle are enlarged in the yellow square.

During the first experiment, five different genotypes were measured every second day from day 14 after planting until day 42 after planting. From day 15 after planting until day 29 after planting combined drought and heat stress was applied. The plants had between three and nine tubers which shows that the algorithm can segment the tubers even if the space between the tubers is restricted. In Figures 5A–E the growth analysis for the genotypes Agria, Saturna, Tomensa, Diamant and Ramses is shown, respectively. The subfigures represent the growth of the tubers for one plant of the respective variety, exemplary. Each curve is related to one tuber of this particular potato plant. The growth curves clearly show that during the drought and heat stress the increase of volume is slower than in the recovery phase. Each genotype basically exhibited the same growth dynamic: In the first growth phase, tubers grew until stress treatment was started. In the second growth phase, after commencing the stress treatment, tuber growth slowed down and eventually stopped. In the last growth phase, after the stress was relieved, tubers resumed their growth. An unusual observation is that after the stress period there was at least one tuber which didn’t continue growing. This was seen for all plants which had more than three tubers before stress application (Figures 5A–E).

**FIGURE 6:**
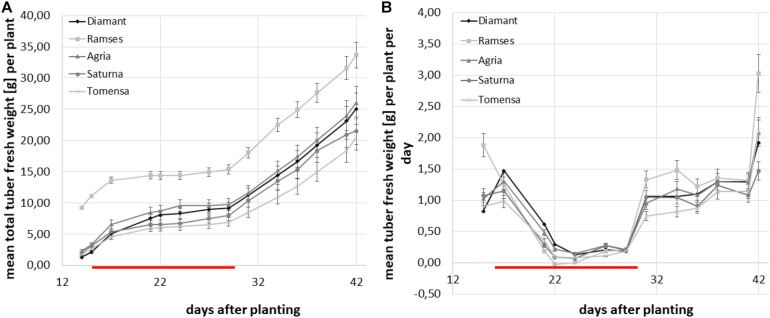
Average total tuber mass per plant and genotype over the experimental time-course. Plants were grown in the greenhouse until tuber induction and then transferred to phytochambers under long day conditions. The red line indicates the period of elevated temperature and drought from day 15 to day 29. Tuber growth was monitored three times per week in four plants of each of the cultivars; Diamant (diamond), Ramses (quadrat), Agria (triangle), Saturna (circle), Tomensa (cross). Error bars represent standard deviations of four biological replicates. **(A)** Average total tuber biomass per plant over the experimental time course; **(B)** average growth velocity of total tubers per plant in g/d.

Additionally, the total tuber biomass was calculated for each plant individually as the sum of all tuber weights of the respective plant at each time-point and the average total tuber fresh weight was calculated for all plants of the same cultivar. For all genotypes analyzed, the increase of total tuber biomass stagnated during the combined abiotic stress treatment between days 15 and only very small increments in tuber biomass were observable (Figure 6B). After the stress treatment, tubers resumed growth in all cultivars. As shown in Figure 6A the resulting pattern of tuber biomass accumulation thus exhibited a bi-phasic course.

### Comparison of Tuber Growth Under Combined Heat and Drought Stress and Control Conditions

**FIGURE 7:**
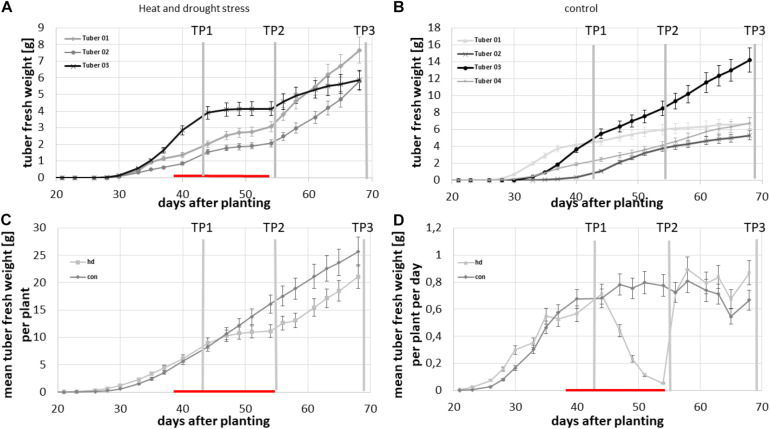
Growth characteristics of potato tubers of cv. Diamant under normal plant growth conditions (con) and under combined heat and drought stress (hd). **(A)** Growth of each tuber of one plant of the stressed group, **(B)** growth of each tuber of one plant of the control group, **(C)** average total tuber fresh weight per plant over the experimental time course, and **(D)** average growth velocity of total tubers per plant in g/d. Error bars represent standard deviations of five to eight plants per treatment.

To investigate whether the bi-phasic growth curve is a response to the combined heat and drought stress, a second experiment with only one genotype but with a control group was conducted. Therefore, thirty potato plants of the cultivar Diamant were grown in two phytochambers. Initially, the same temperature regime was applied to all plants until tubers developed i.e., 16 h light at 21°C and 8 h dark at 18°C and 50% humidity during the day and 35% humidity at night. *Via* the CT analysis pipeline, we monitored the tuber growth three times per week in 16 pots and utilized the same calibration set (see Figure 3) as in the previous experiment to estimate the individual tuber biomass from the volumetric data. The stress treatment was applied when all plants exhibited detectable tubers. After this, we applied to half of the plants a combined heat and drought stress by increasing ambient temperature to 29 °C during the day and 21°C during the night for 2 weeks and simultaneously decreasing daily water supply to 30 ml per day/plant instead of 50 ml per day/plant for control conditions. A few days after commencement of the stress treatment, tubers in the stressed group of plants ceased growing (Figure 7A) while tubers in the control group continued their growth (Figure 7B). Combined stress treatment led to a discontinuation of growth of all tubers, which is visible in Figure 7A, where all growth curves become flat. Control tubers showed constant growth although at varying velocities, as depicted in Figure 7B for tubers of one exemplary plant. This became clearly visible when average total tuber biomass per plant was calculated for each condition (Figure 7C) and, moreover, when the average biomass increase per day per plant was investigated (Figure 7D). Four days after the beginning of the stress treatment, a decrease in growth velocity was already measurable and it dropped close to zero at the end of the stress period. In contrast, tubers of plants kept under control conditions showed constant growth, which is depicted for the mean tuber biomass of monitored plants in Figure 7D. After releasing the plants from the stress treatment, growth velocity immediately increased to a level similar to that of the control treated plants resulting in a bi-phasic growth pattern (Figures 7C,D).

### Abiotic Stress Marker Gene Expression Analysis Confirms Combined Stress Treatment

To validate that the stress treatment had an effect on tuber physiology, the expression of potential marker genes for stress were investigated. Therefore, tuber samples taken 8 days after commencement of the combined stress treatment (TP1), 3 days after cessation of stress treatment (TP2) and after a 14-day recovery phase at the end of the experimental period (TP3) were subjected to qRT-PCR analysis. Stress-responsive genes were selected from publications on drought stress and subsequent re-watering in potato stolons (Gong et al., 2015), potato plants exposed to elevated temperatures (Hancock et al., 2014) and combined heat and drought stress in tobacco (Rizhsky et al., 2002).

**FIGURE 8:**
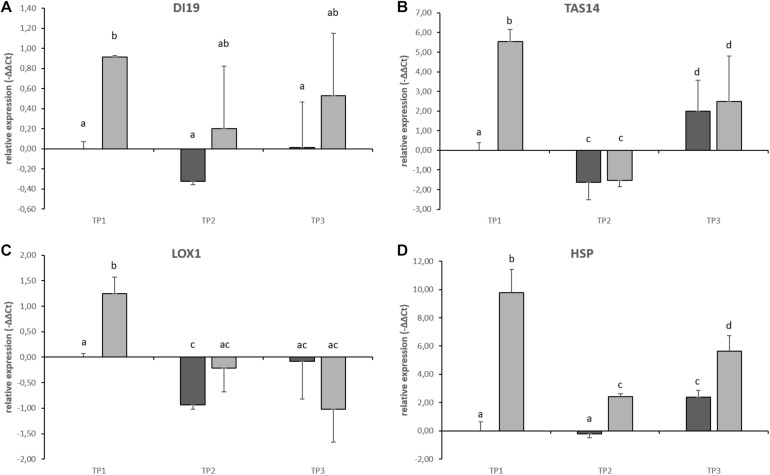
qRT-PCR on stress-responsive gene expression in tuber samples of the potato cultivar Diamant. Results from control plants are shown in dark gray, stress treated plants are shown in light gray. Error bars represent standard deviation of three to four biological replicates. **(A)** Drought-induced 19 (DI19), **(B)** Abscisic acid and environmental stress-inducible protein (TAS14), **(C)** Lipoxygenase 1 (LOX1), and **(D)** Heat-shock protein (HSP). Different subscript letters indicate statistically different expression (*p* < 0.05).

Gene expression of candidate stress marker genes drought-induced 19 (*DI19*), Abscisic acid and environmental stress-inducible protein (*TAS14*), Lipoxygenase (*LOX1*), and Heat-shock Protein (*HSP*) was significantly increased during the heat treatment at TP1 when compared to potato tubers grown under control conditions (Figures 8A–D). After relieving the stress from the plants, gene expression of DI19, *TAS14* and *LOX1* decreased to control values (Figures 8A–C, TP2 and TP3). The only potential stress marker gene whose expression did not return to control values was a *HSP* whose expression stayed significantly above control values until the end of the experimental period although to a lesser extent as during the stress treatment at TP1 (Figure 8D). The expression of *DI19* was significantly increased during the stress treatment but showed a lot of variation after stress release at TP2 and 3 (Figure 8A) indicating that individual tubers adjust differently to the changed environmental conditions.

### Impact of Combined Stress on Starch Metabolism

**FIGURE 9:**
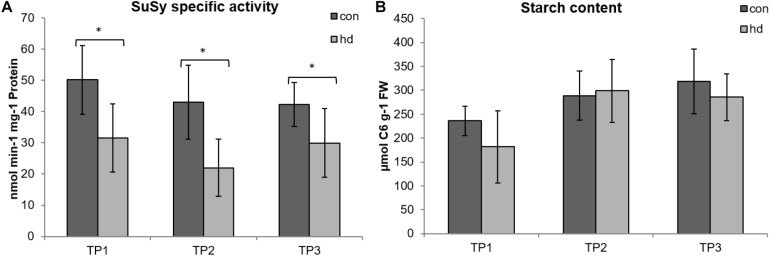
SuSy-activity **(A)** and starch content **(B)** of tubers from plants grown under control and combined stress conditions. Dark gray bars represent values from control treated plants; light gray values represent values from stressed plants. Error bars represent standard deviations of nine to 10 biological replicates and six to 10 biological replicates for SuSy and starch measurements, respectively (*p < 0.05, Student’s *t*-test).

Abiotic stress is known to cause yield penalties and quality loss in potato (Levy, 1985, 1986). Since starch content is the main factor determining potato tuber dry matter and an important trait for breeders (Li et al., 2008), the impact of the stress treatment on starch metabolism was investigated. Therefore, activity of sucrose synthase, a marker enzyme for starch biosynthesis (Zrenner et al., 1995; Baroja-Fernández et al., 2009) was measured. In comparison to the plants grown under control conditions, SuSy-activity was decreased during the stress treatment and remained low throughout the rest of the experimental period (Figure 9A). In contrast, when measuring starch contents of the tubers at the three time-points, no significant changes were detected (Figure 9B).

**FIGURE 10:**
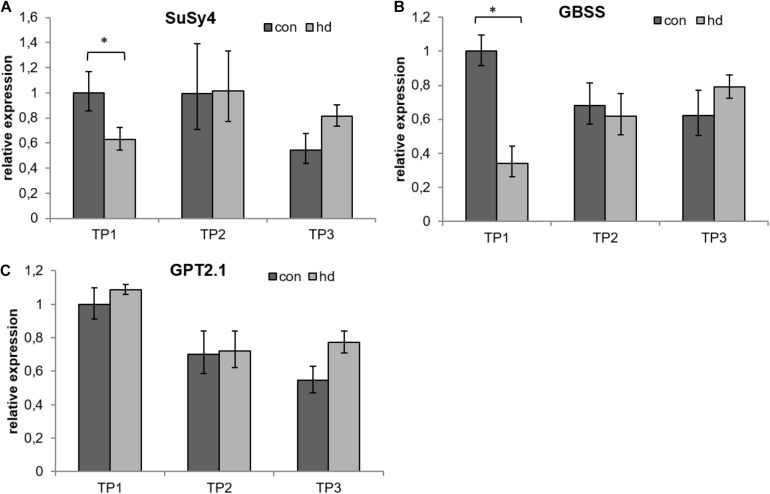
Relative expression of marker genes for starch biosynthesis. **(A)** Sucrose synthase 4 (*SuSy4*), **(B)** Granule-bound starch synthesis (*GBSS*), and **(C)** Glucose-6-phosphate translocator 2.1 (*GPT2.1*). RNA expression was assessed by qRT-PCR with specific primers and normalized to ubi3 expression by 2^–ΔΔ*Ct*^ method. Values of control treated plants are shown in dark gray; stressed plants are shown in light gray. Error bars represent standard deviation of four biological replicates. *p < 0.05 (Student’s *t*-test).

To further elucidate the impact of the stress treatment on starch metabolism, gene expression of *SuSy4*, the main SuSy isoform in potato tubers (Fu and Park, 1995; Van Harsselaar et al., 2017) and a determinant of sink strength (Zrenner et al., 1995) was evaluated *via* qRT-PCR analysis. During the stress treatment (TP1) a marked decrease in *SuSy4* expression in comparison to the control group was detected (Figure 10A). After the stress treatment (TP2) *SuSy4* expression recovered to control level. At the end of the experimental period (TP3) *SuSy4* expression in stress treated tubers exceeded the expression in control tubers Figure 10A). As a second starch metabolism marker, the expression of granule-bound starch synthase (*GBSS*) was analyzed. Similar to *SuSy4*, *GBSS* expression was found to be significantly down-regulated in tubers of the stressed group during the stress treatment (TP1) and to recover to control values after the stress (TP2 and 3, Figure 10B). Additionally, GPT2.1 expression was analyzed. During the stress treatment and 1 week after ending the treatment, no effect of the stress on *GPT2.1* expression could be observed. At the end of the experimental period (TP3), *GPT2.1* expression was slightly, but non-significantly higher in tubers grown under stress conditions than in tubers grown under control conditions (Figure 10C).

In summary, analysis of stress markers as well as markers for starch biosynthesis show that the biphasic growth curve is due to the combined drought and heat stress response which affects tuber biomass accumulation. Although starch content was not significantly affected by the stress treatment, biochemical and molecular biological parameters suggest an impact on tuber metabolism which is in line with the observation that tuber growth was inhibited by the stress treatment (Figure 7).

## Discussion

### Combined Heat and Drought Stress Affects Tuber Growth

In this study, potato tuber development from initiation until harvest was monitored by CT analysis in stressed plants and plants grown under ambient conditions. We were able to establish an imaging pipeline to analyze the potato tuber delevopment in a high thoughput (5 min per pot) and automated way (one set of parameter for the whole experiment). This enabled analyzing the stress dependend growth dynamic of individual tubers within one pot and to visualize even small variations in belowground biomass. The time dependance of this data set allowed the 4D analysis of growth processes affected by abiotic stresses. The imaging pipeline presented here can be scaled up to be used in high-throughput phenotyping systems. However, the combination with automated data processing is the key to generate objective data accelerating breeding efforts to improve abiotic stress tolerance.

We chose combined heat and drought stress since these abiotic stresses are likely to occur in parallel in the context of global climate change (Hijmans, 2003; Ahuja et al., 2010). We applied the stress treatment after tuber induction, when tubers were detectable by CT, in order to avoid delaying or even completely inhibiting tuber formation. Such detrimental effects of heat and drought have been described previously (Jackson, 1999; Luitel et al., 2015; Dahal et al., 2019; Aliche et al., 2020). During combined stress application, CT analysis revealed that tuber growth is inhibited under combined elevated temperature and drought stress and most of the tubers can resume after the stress has been terminated. The growth arrest as calculated from the CT data led to significantly lower tuber biomass in stressed plants at the end of the stress treatment compared to control plants. After cessation of the stress, tubers started growing again and were only slightly smaller than control tubers at the end of the experimental period.

Due to the early timing of the stress treatment directly after tuber induction, it seems that most tubers were able to recover from the implications of the stress treatment on biomass accumulation. This was also seen in an early study on the effects of individual heat and drought stress on tuber development (Levy, 1985) where early stress, imposed when tubers were small, reduced tuber yield and dry matter accumulation only slightly. Heat or drought stress applied during the tuber bulking stage had a more deleterious effect on tuber yield (Levy, 1986, 1985). Moreover, in our experimental setup plants were still immature at the end of the experimental period, exhibiting only small tubers and low overall tuber biomass. How our findings translate to potato plants grown to maturity requires further trials.

### Stress Markers Respond to the Treatment

Genes which have previously been shown to respond with differential expression during abiotic stress were selected from the literature (Rizhsky et al., 2002; Gong et al., 2015) in order to confirm that the stress led to a response in the potato tubers. Gong et al. (2015) analyzed gene transcription in stolon tips of potato plants grown under control conditions, drought stress and after re-watering. They found that *TAS14* was 4.7-fold up-regulated after 3 days of drought treatment and 8.2-fold down-regulated after re-watering when compared to stolons from plants grown under control conditions. In our study, *TAS14* expression in tubers was almost 50-fold up-regulated after 8 days of combined drought and heat stress but had returned to values similar to control 3 days after stress release. This indicated that *TAS14* might be suitable as a marker for stress in potato tubers. However, further characterization of its expression profile in different plant organs and under differing conditions is needed to confirm its suitability as a stress marker. Support for the role of TAS14 during abiotic stress comes from experiments in tomato, where stable overexpression of *TAS14* led to improved long-term drought tolerance (Muñoz-Mayor et al., 2012).

Rizhsky et al. (2002) examined gene expression patterns under different stress conditions as well as their combinations in tobacco plants. A combination of drought and heat stress led to significant increases in gene expression of *DI19* and *Lox1*, by 34- and 6.7-fold, respectively. *DI19* has also been described in rice as a key regulator during drought stress and drought tolerance (Wang et al., 2014). In the present study, *DI19* and *Lox1* were induced significantly, but to a far lesser extent than in those previous studies, in tubers during combined stress treatment compared to tubers grown under control conditions. It appears that these two transcripts are not suitable as markers for combined heat and drought stress in potato tubers.

Heat-shock protein (DMT400032851) which was strongly elevated in potato tubers during combined heat and drought stress compared to control, has previously been found in a microarray analysis among 2,886 differentially expressed genes in potato tubers of the cultivar Desirée during mild heat (Hancock et al., 2014). In the experiment by Hancock et al. (2014), potato plants were subjected to elevated temperature (30°C during the day / 20°C during the night) for 1 week and expression patterns over a time course of 20 h were compared to tubers grown under ambient conditions (22°C / 16°C). *HSP* was found to be upregulated approximately 12-fold on average over time (range 0.8–30.8-fold) during elevated temperature. The strong induction which we have determined for *HSP* could be a result of the additional drought treatment and the different methodology (qRT-PCR vs. microarray analysis). Thus, *HSP* might be an appropriate marker for combined heat and drought stress in potato tubers but further validation is recommended.

### Combined Heat and Drought Stress Has a Negative Influence on Expression of Genes Encoding Enzymes Involved in Starch Biosynthesis

Heat and drought are abiotic stress factors influencing many developmental and physiological processes. In potato plants, both factors, alone or in combination, affect tuberization and starch accumulation associated therewith (Bodlaender, 1963; Wolf et al., 1990; Gawronska et al., 1992). Depending on the timing of the occurrence of these disruptive environmental conditions, tuberization can be inhibited completely or tuber bulking can be disturbed (Tang et al., 2018). Furthermore, carbon partitioning can be altered by transient exposure of potato plants to heat stress leading to reduced starch and increased reducing sugar contents of tubers (Busse et al., 2019). We have seen a disturbance of tuber bulking which was confirmed by analysis of mRNA expression of *SuSy4* as well as specific activity of SuSy, a marker for starch biosynthesis, in tuber samples. Increased *SuSy* expression and activity has been associated with increased starch and total yield (Baroja-Fernández et al., 2009). Under adverse conditions like heat, *SuSy4* expression and SuSy activity have been shown decrease (Hastilestari et al., 2018).

GPT2.1 has been identified as the tuber-specific GPT2 isoform (Van Harsselaar et al., 2017), whose expression is strongly associated to processes linked to starch biosynthesis and correlates to *SuSy4* expression (Ferreira et al., 2010). Therefore, we hypothesized that *GPT2.1* expression would decrease during stress treatment. However, gene expression analysis of *GPT2.1* revealed no significant differences between stressed tuber samples and tubers grown under control conditions. This is consistent with the gene expression data from tuber samples under elevated temperatures published by Hancock et al. (2014) and Hastilestari et al. (2018) where *GPT2.1* was not among the differentially regulated genes.

Granule-bound starch synthase is the starch synthase isoform responsible for amylose-synthesis (Visser et al., 1989). Expression of *GBSS* was found to be significantly down-regulated in potato tuber during combined heat and drought stress in our qRT-PCR analysis. Similarly, in the microarray experiment by Hancock et al. (2014), *GBSS* expression was downregulated significantly in the tuber samples from plants grown under elevated temperature. This seems consistent with an overall decrease of starch biosynthesis in potato tubers under heat and drought stress. In our experiment, *SuSy4* and *GBSS* expression recovered to levels of tubers grown under control conditions after the stress conditions were released. Similar observations were reported by Chen et al. (2020) in potato leaves during re-watering after a dehydration period, where most genes which were differentially expressed during the dehydration period reversed their expression during re-watering.

### CT Analysis Pipeline Is a Phenotyping Tool for Stress Detection

The presented imaging pipeline was able to generate stable results in growth analysis of potato tubers for five different genotypes. Using only one set of parameters it was possible to track individual tubers for different growth stages and a high variation in tuber number per plant. Thus, we demonstrated that this kind of CT analysis is a suitable tool to monitor morphological changes of otherwise inaccessible, underground tissues *in planta* and enables also methods like guided sampling to correlate in future morphological and physiological measurements. This is in accordance with for example Pfeifer et al. (2015) or Metzner et al. (2015). With the presented image pipeline for segmentation and feature calculation it is possible to track each individual potato tuber over time within the experiment. These time-resolved data were used for more detailed growth curve analysis like non-invasive biomass determination and the assessment of growth velocity. We could show that the observed biphasic growth pattern is directly connected with the heat and drought stress response in the tubers. This enabled us to use this CT based method together with the presented image pipeline to be a non-invasive tool for prediction of stress in potato tubers already in early growth stages. A major point was the presented combination of the individual 3D volume image analysis techniques. Doing so we could use only one set of input parameters for the extraction of the individual tubers for the whole experiment. Thus, the volume analysis toolchain was robust enough to handle the individual noise in the reconstructed volumes and the differences in moisture between well-watered and heat and drought stressed plants. For potato breeding purposes, this method could be used as a phenotyping platform for the development of stress resistant varieties. Additionally, the robust image pipeline and the relatively fast measurement times enables the observation of below ground tuber growth in high throughput. For future experiments it is possible to use pots up to a diameter of 20 cm and a volume of 4 L to analyze potato plants also in later growth stages. However, a deeper understanding of the processes and regulatory circuits elicited by drought, heat or both combined is required for potato in order to define parameters to distinguish stress tolerant and susceptible varieties. This is an important factor toward more resistant genotypes.

In general, the throughput of the system has to be evaluated in terms of cycle time in the measurement—including the measurement time and the time for sample exchange—and the time for data processing. The data processing time is directly connected to the computing power used for the analysis and with the data analysis pipeline presented a parallel computing can be realized. Thus, the processing time is not a bottleneck to further increase the overall throughput of the combined system. However, the current system was relying on a manual change of the individual plants. To further increase the cycling time of the system an integration in a conveyor system would be highly beneficial. Doing so, the time for sample exchange could be minimized. To decrease the measurement further, there are some more steps possible. The easiest one is to decrease the resolution and at the same time increase the smallest detectable tuber. This option is strongly connected with the breeding use case in mind. If this is not an option, using stronger X-ray sources or even pulsed sources is a way to decrease the scanning time. Finally, a vertical gantry system with pulsed X-ray sources with rotating anodes would be a possibility for real high-throughput tuber analysis.

## Data Availability Statement

The raw data supporting the conclusions of this article will be made available by the authors, without undue reservation.

## Author Contributions

JC, SG, and JV performed the experiments and drafted the manuscript. JV did the molecular biological work. SG supervised and together with JC performed the CT work. NW and JC performed the image and data analysis. US and NU provided the project funding, conceived and led the study, and contributed to writing the manuscript. US, SG, and JL discussed the work and the biphasic growth pattern. All authors read and approved the final manuscript.

## Conflict of Interest

The authors declare that the research was conducted in the absence of any commercial or financial relationships that could be construed as a potential conflict of interest.
